# Family Experiences of the Strong Families Program in Thailand: Prospective Mixed Methods Cohort Study

**DOI:** 10.2196/98447

**Published:** 2026-09-08

**Authors:** Nida Buawangpong, Jacqueline E Hackett, Pasiri Singhasiri, Nopakoon Nantsupawat, Pimpisa Chomsri, Chaisiri Angkurawaranon, Apinun Aramrattana, Amalee McCoy, Erika Lisette Roach, Wachiranun Sirikul, Wichuda Jiraporncharoen

**Affiliations:** 1 Department of Family Medicine, Faculty of Medicine, Chiang Mai University, 110, Intrawarorot road, Sriphum, Meaung, Chiang Mai, 50200, Thailand, 66 53935462; 2 Global Health and Chronic Conditions Research Center, Chiang Mai University, Chiang Mai, Thailand; 3 Fulbright-Fogarty Fellow in Public Health, Faculty of Medicine, Chiang Mai University, Chiang Mai, Thailand; 4 Department of Health Policy and Management, Johns Hopkins Bloomberg School of Public Health, Maryland, MD, United States; 5 School of Nursing, Mae Fah Luang University, Chiang Rai, Thailand; 6 Department of Psychology, University of California Berkeley, California, CA, United States; 7 Department of Community Medicine, Faculty of Medicine, Chiang Mai University, , Sriphum subdistrictChiang Mai,, Thailand

**Keywords:** parenting skills, positive parenting, family health, program evaluation, mixed methods

## Abstract

**Background:**

The Strong Families (SF) Program, developed by the United Nations Office on Drugs and Crime, is an evidence-informed family skills intervention designed to support caregivers and children in low-resourced, high-stress circumstances. Following a pilot phase, we revised the program materials according to the expert trainers’ suggestions to best reflect the needs of the target population.

**Objective:**

This study aimed to evaluate the feasibility, effectiveness, and cultural adaptability of the second phase of the SF Program expansion in northern Thailand.

**Methods:**

This prospective mixed methods pretest-posttest feasibility cohort study involved 68 families (caregivers and children as 1:1) from Chiang Mai, Lamphun, and Chiang Rai—3 provinces in northern Thailand. The applied intervention consisted of a 3-week program with both separate and joint sessions for caregivers and children. Quantitative data were collected using the Parent and Family Adjustment Scales, Strengths and Difficulties Questionnaire, and Child and Youth Resilience Measure–Revised–Person Most Knowledgeable About the Child. Data were analyzed using linear mixed-effects regression. Qualitative data were gathered through semistructured interviews with 19 families and analyzed using content analysis to identify key themes.

**Results:**

Quantitative analysis using linear mixed-effects regression revealed significant improvements in coercive parenting (β=−0.10; *P*=.003), child conduct problems (β=−0.04; *P*=.002), and peer relationship problems (β=−0.04; *P*=.048) for parents and in emotional (β=–0.05; *P*=.01) and conduct (β=−0.04; *P*=.02) behavior for children. However, scores for positive encouragement (β=0.05; *P*=.009) and parent-child relationship (β=0.28; *P*<.001) significantly declined, a discrepancy that may be due to response shift bias, in which participants adopt stricter self-evaluation standards after the intervention. Qualitative findings showed 5 themes that highlighted improved family atmosphere, better emotional regulation, and successful translation of knowledge into practice to better develop protective factors for young people. Participants viewed the program as feasible and culturally appropriate but recommended extending the duration and increasing paternal involvement for more significant, sustainable outcomes.

**Conclusions:**

The SF Program is a culturally adaptable and effective intervention for improving family dynamics in low-resource settings.

## Introduction

Families worldwide face a range of adversities that undermine their capacity to provide safe and nurturing environments for children. These challenges include unemployment, schooling barriers, and limited access to goods and services in rural and underserved areas [1], as well as migration, displacement, residence in refugee camps, and exposure to conflict or postconflict situations [2]. Such circumstances place significant strain on caregivers and compromise their ability to engage in responsive, supportive parenting. Children’s earliest and most formative interactions occur within the family, well before they reach school age, and those whose caregivers lack nurturing abilities, parenting skills, or the capacity to manage stressors such as poor health or financial hardship are at heightened risk of maladjustment across cognitive, emotional, and behavioral domains [3,4]. Strengthening family skills and competencies is therefore essential not only to support caregivers but also to foster the safe, stable, and nurturing relationships that promote children’s healthy development and the well-being of both caregivers and children [5].

The United Nations Office on Drugs and Crime (UNODC) has implemented evidence-based family skills prevention programs in low- and middle-income countries worldwide since 2010 [6]. In 2017, the Strong Families (SF) Program was developed [7]. The program is designed for families in underserved settings, offering an evidence-informed prevention response that builds family skills to benefit the health and safe development of children. This program can be easily adapted to serve families in different contexts facing adverse circumstances. SF helps caregivers and children manage daily stressors and difficulties, aiming to strengthen family structures and functions to prevent drug use, violence, and other negative social consequences in their children. It is a universal program within this selective subgroup of families and is best suited for families with children aged between 8 and 15 years [7].

More than 30 countries have implemented the SF Program and found it effective in improving family relationships and parenting skills. SF was initially created and tested in Afghanistan and has since been implemented in Central America, Central and West Asia, and East and West Africa. An early 2024 pilot study of SF in Thailand was evaluated and refined based on the results of a previous project [8]. SF was found to be feasible to implement in Thailand during this first phase. The program strengthened family relationships and improved mutual understanding among family members. However, several challenges were identified, including language barriers, cultural mismatches in the materials, and difficulty handling challenging situations arising during program facilitation. In phase 2 (late 2024), several materials were adapted for the target population. Revisions included using simpler Thai words, Asian faces, and pictures that were easy to understand in the Thai cultural context. Training sessions for trainers, including skills to offer brief interventions, advice, and strategies for handling difficult situations during program implementation, were incorporated into program preparation. Therefore, the objective of this study was to evaluate the program’s feasibility, effectiveness, and adaptability for further expansion in the country.

## Methods

### Study Design

**Figure 1.**
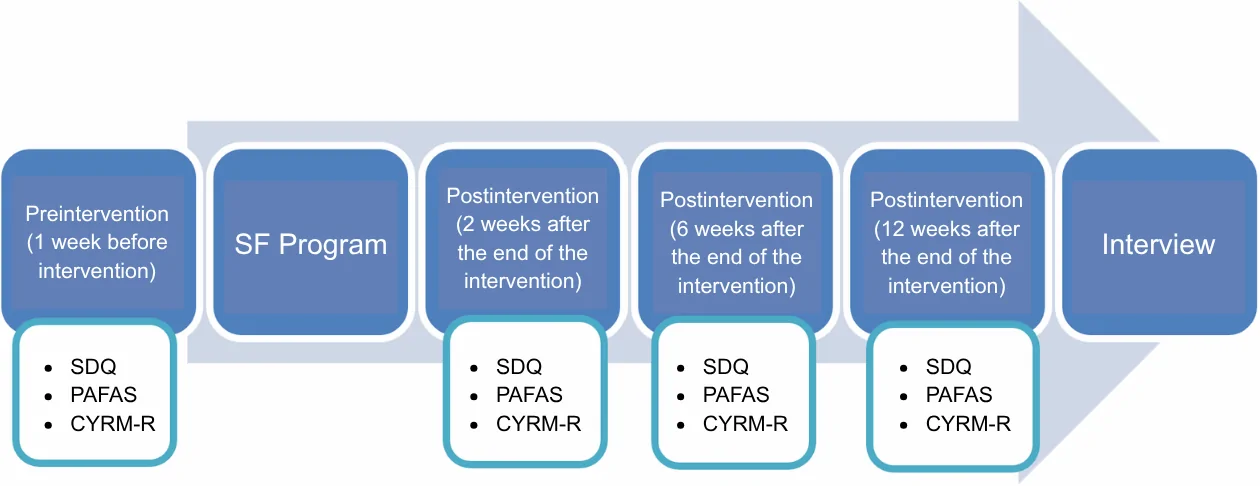
Study timeline. CYRM-R: Child and Youth Resilience Measure–Revised; PAFAS: Parent and Family Adjustment Scales; SDQ: Strengths and Difficulties Questionnaire; SF: Strong Families.

This study used a mixed methods, pretest-posttest feasibility design with a prospective cohort of participants enrolled in the SF Program. Phase 2 ran a follow-up data collection at 4 time points (before the intervention and at 2, 6, and 12 weeks after the end of the intervention). The pretest was completed prior to the first week, and the posttest was completed within 12 weeks of the final programming date for estimating the long-term outcomes. Additional qualitative interviews were conducted between August 2025 and September 2025, approximately 12 weeks following the last session. Figure 1 illustrates the study timeline.

### The SF Program

This program was conducted between June 2025 and July 2025 in the Chiang Mai, Chiang Rai, and Lamphun provinces in northern Thailand, encompassing 8 study locations in total. Training for the Trainers of the SF Program was a 3-day workshop for trainers who would conduct SF in their local geographic areas. The workshop was led by master trainers certified by UNODC. The SF Program consisted of 3 sessions held on 3 consecutive weekends engaging caregivers and children. The first session was exclusively for caregivers, whereas children participated in parallel and joint activities in the second and third sessions, respectively. The topics for caregivers were (1) understanding strengths and stressors, (2) using love and limits, and (3) teaching children what is right. The topics for children were (1) learning about stress and (2) following rules and appreciating parents. The topics for joint sessions were (1) learning about each other and (2) supporting values and dreams. To ensure implementation fidelity, UNODC-certified master trainers attended and provided feedback for the Thai trainers at community sites during the first session of the SF Program.

### Participants

The target population for this study consisted of the families (specifically, the caregivers and their children) who participated in the SF Program phase 2. We used a convenience sampling strategy recruiting 68 volunteer families (caregiver-child dyads) from 8 study locations across the 3 provinces. Families with children aged 8 to 15 years were included, aligning with the program’s target demographic.

### Sample Size Calculation

#### Quantitative Study

Using data from the first SF Program in Afghanistan [7], the total difficulty scale before and after completing the program was reported as 17.77 (SD 5.67) scores and 10.62 (SD 4.98) scores, respectively. The sample size calculation was based on a 1-group repeated-measure ANOVA [9,10] using the *wp.rmanova* package in R (R Foundation for Statistical Computing), a moderate effect size (*f*=0.25), a correlation of 0.5, power of 80%, and an α of .05. The required sample size was at least 45 participants to detect the effectiveness of the SF Program.

#### Qualitative Study

This project in Thailand included a sample size of 19 families (caregiver-child dyads), approximately 2 to 3 families per site across 8 locations in the 3 provinces. Volunteer families from each site were included. In the previous project with SF Program trainers, data saturation was achieved with interview data from 10 participants [8].

### Ethical Considerations

This study was conducted following review and approval from the Institutional Ethics Committee of the Faculty of Medicine, Chiang Mai University, Chiang Mai, Thailand (ethics approval number 143/2025). All participants provided written informed consent prior to study commencement in accordance with the 1964 Declaration of Helsinki.

### Data Measurement and Collection

#### Quantitative Study

Quantitative data were collected using the following validated instruments. The Parent and Family Adjustment Scales (PAFAS; 30 items) assessed caregivers’ perceptions of their parenting practices, family functioning, and personal adjustment. The Strengths and Difficulties Questionnaire (SDQ; 25 items) assessed caregivers’ perceptions of their children’s emotional symptoms, conduct problems, hyperactivity, peer problems, and prosocial behavior. The Child and Youth Resilience Measure–Revised–Person Most Knowledgeable About the Child (CYRM-R-PMK; 17 items) assessed both caregivers’ perceptions of their children’s resilience and coping and children’s self-reported resilience.

#### Qualitative Study

Qualitative data were gathered prospectively after the intervention through semistructured interviews guided by the reach, effectiveness, adoption, implementation, and maintenance framework [11,12]. Interviews with volunteer families were conducted individually, one family at a time. All participants were informed of the research study and provided consent with permission for audio recording. Each interview lasted approximately 40 minutes and was conducted on the same site as the workshops by research assistants who were not involved in the SF Program, supervised by NB and WJ.

### Data Analysis

#### Quantitative Study

Descriptive statistics were used to summarize participant characteristics. The scoring key for the PAFAS indicates that higher scores reflect lower levels of parenting skills and family adjustment [13]. For the SDQ, higher scores indicate greater problems in emotional regulation, conduct, restlessness, and peer relationships; however, higher scores in the social relationships domain (prosocial behavior subscale) indicate greater strengths [14]. For the CYRM-R-PMK, higher scores reflect higher levels of child and youth resilience [15]. To examine the change in family functions in each domain of the CYRM-R-PMK, PAFAS, and SDQ over time (a week), a linear mixed-effects regression model was applied. The scores were treated as a continuous dependent variable. Time (week) was analyzed as a fixed effect. Child age, child sex, parent age, and parent sex were adjusted as potential confounders [16,17]. To account for within-subject correlation due to repeated measurements, a random intercept was specified for each participant. Model parameters were estimated using restricted maximum likelihood. Regression coefficients with corresponding 95% CIs were reported. All analyses were performed using Stata (version 17; StataCorp), and statistical significance was set at *P*<.05.

#### Qualitative Study

The qualitative interview data were analyzed using content analysis to evaluate the overall program. The datasets were uploaded into NVivo (version 12; Lumivero). Each transcript was read multiple times to aid familiarization and check the accuracy of each transcript. Two independent researchers (NB and WJ) led the analysis. Then, preliminary results were discussed with all authors. The inductive coding was developed from patterns in the text data and adapted iteratively during analysis. The identified codes were compared, and the similarities and differences were discussed until a consensus on the emergent themes and subthemes was reached.

## Results

### Overview

A total of 68 families attended the SF Program across the Chiang Mai, Lamphun, and Chiang Rai provinces in 2025. Most caregivers (59/68, 86.8%) were female, with a mean age of 47.4 (SD 13.8) years. In total, 51.5% (35/68) of the children were male, and their mean age was 10.8 (SD 1.6) years.

Table 1 shows the scoring and changes in all assessment tools. Regarding the PAFAS, the results showed a significant improvement in coercive parenting (β=–0.10; *P*=.003) as the decrease in scores reflected a reduction in maladaptive behaviors. However, the analysis indicates a significant increase in scores for positive encouragement (β=0.05; *P*=.009) and parent-child relationship (β=0.28; *P*<.001); based on the inverted scoring key, these suggest a decline in positive parenting skills and relationship quality during the study period. In terms of child difficulties (evaluated using the SDQ), parents reported significant improvements, evidenced by decreases in conduct problems (β=−0.04; *P*=.002) and peer relationship problems (β=−0.04; *P*=.048). However, social relationships (a strength-based domain) showed a decrease in scores (β=−0.05), suggesting a reduction in strengths, although this was not statistically significant (*P*=.09). Similarly, children self-reported significant improvements in emotional (β=−0.05; *P*=.01) and conduct (β=−0.04; *P*=.02) behaviors. No substantial changes were observed in child resilience (CYRM-R-PMK) from either the parent or child perspectives.

**Table 1.** Scoring and changes in all assessment tools.

Measurements	Pretraining time point, mean (SD)	2 weeks after training, mean (SD)	6 weeks after training, mean (SD)	12 weeks after training, mean (SD)	β coefficient (95% CI)^a^	*P* value
Parents						
PAFAS^b^						
Parenting skills						
Parental consistency (0-15)	6.16 (1.96)	6.12 (2.31)	5.68 (2.76)	6.00 (2.39)	−0.02 (–0.07 to 0.03)	.48
Coercive parenting (0-15)	5.88 (3.13)	5.12 (2.83)	4.87 (2.99)	4.53 (2.83)	−0.10 (–0.16 to 0.03)	.003
Positive encouragement (0-9)	2.12 (1.93)	2.23 (2.04)	2.07 (1.74)	2.79 (1.38)	0.05 (0.01 to 0.09)	.009
Parent-child relationship (0-15)	1.74 (1.97)	2.43 (2.70)	1.79 (2.40)	5.37 (1.79)	0.28 (0.23 to 0.33)	<.001
Family adjustment						
Parental adjustment (0-15)	6.03 (2.09)	5.71 (2.38)	5.25 (2.48)	6.46 (2.24)	0.04 (–0.02 to 0.09)	.16
Family relationships (0-12)	2.76 (2.10)	2.40 (1.93)	2.21 (1.98)	3.31 (1.48)	0.05 (0.01 to 0.09)	.02
Parental teamwork (0-9)	2.37 (2.10)	2.37 (2.10)	1.50 (1.77)	2.16 (1.91)	−0.03 (–0.06 to 0.01)	.14
SDQ^c^						
Emotional behavior (0-10)	2.99 (1.87)	2.51 (1.80)	3.22 (2.14)	2.28 (2.03)	−0.04 (–0.08 to 0.01)	.09
Conduct behavior (0-10)	1.81 (1.43)	1.47 (1.37)	1.53 (1.59)	1.21 (1.30)	−0.04 (–0.07 to –0.01)	.002
Restless behavior (0-10)	3.53 (2.28)	3.04 (2.25)	3.06 (2.27)	2.93 (2.27)	−0.04 (–0.08 to 0.01)	.07
Peer relationship (0-10)	3.03 (1.41)	2.62 (1.60)	2.71 (1.81)	2.47 (1.38)	−0.04 (–0.07 to –0.01)	.048
Social relationship (0-10)	7.56 (1.83)	6.62 (2.38)	6.26 (2.79)	6.79 (2.32)	−0.05 (–0.10 to 0.01)	.09
CYRM-R-PMK^d^						
Child and youth resilience (17-85)	73.71 (7.11)	67.43 (18.40)	64.62 (23.12)	69.01 (16.70)	−0.06 (–0.19 to 0.07)	.37
Children						
SDQ						
Emotional behavior (0-10)	3.96 (2.17)	3.54 (1.86)	3.22 (2.14)	3.28 (2.10)	−0.05 (–0.09 to –0.01)	.01
Conduct behavior (0-10)	2.53 (1.71)	2.49 (1.63)	2.22 (1.66)	2.12 (1.49)	−0.04 (–0.07 to 0.01)	.02
Restless behavior (0-10)	3.93 (1.96)	3.96 (2.23)	3.15 (2.23)	3.66 (2.53)	−0.03 (–0.07 to 0.01)	.11
Peer relationship (0-10)	3.40 (1.71)	2.96 (1.62)	3.03 (1.77)	3.07 (1.68)	−0.02 (–0.05 to 0.02)	.33
Social relationship (0-10)	7.00 (1.65)	6.16 (1.80)	6.46 (2.03)	6.66 (2.01)	−0.01 (–0.05 to 0.04)	.82
CYRM-R-PMK						
Child and youth resilience (17-85)	68.29 (7.12)	64.69 (11.66)	63.85 (16.26)	66.38 (11.41)	0.01 (–0.13 to 0.15)	.92

a Score change per week from the pretraining time point to 12 weeks after training.

b PAFAS: Parent and Family Adjustment Scales.

c SDQ: Strengths and Difficulties Questionnaire.

d CYRM-R-PMK: Child and Youth Resilience Measure–Revised–Person Most Knowledgeable About the Child.

The qualitative study included a total of 19 families, or caregiver-child dyads. The participating caregivers were predominantly female (17/19, 89.5%), comprising 82.4% (14/17) mothers, 11.8% (2/17) grandmothers, and 5.9% (1/17) aunts, whereas the 10.5% (2/19) male participants included 1 father and 1 foundation staff member (caregiver for a child in the foundation). The ages of these caregivers ranged from 28 to 59 years, representing early working age to middle adulthood. Regarding the children, 52.6% (10/19) were female, and 47.4% (9/19) were male, with ages ranging from 7 to 14 years. This age group corresponded primarily to the late primary and early secondary school levels, critical developmental stages (early adolescence and middle childhood) for behavior and responsibility. From the qualitative analysis, 5 themes emerged (Textbox 1).

Textbox 1. **Themes of qualitative results.**

- Theme 1: motivation of program engagement
- Theme 2: perceptions of learning utility
- Theme 3: goodness manifesting across household members
- Theme 4: key facilitators of success
- Theme 5: recommendations for optimization

### Theme 1: Motivation of Program Engagement

The main motivation for enrolling in the program was the desire to improve parenting skills. Caregivers indicated that they wanted to learn the correct ways to teach and manage their children’s behavior and understand their children better. Some caregivers also sought to resolve relationship issues at home, motivated by a desire to reduce family stress or strengthen the bond between mother and children. Other motivations for caregivers and children to participate in the program were varied, including persuasion from community and school leaders; most participants were referred by village heads, schoolteachers, or religious leaders (churches and temples). Finally, some children expressed interest in enrolling so that they could participate in the children’s activities, either as a way for the latter to enjoy time with their parents or as a response to an invitation from friends:

I want to know what “Strong Family” means in terms of strength. I want to know how to raise children properly*.*

[Caregiver; family 7]

I think if we go, we will know how we can talk to children, or how can we be patient with them?

[Caregiver; family 6]

I want to improve my communication skills with my children to reduce the gap.

[Caregiver; family 1]

Mr. Boom from Tambon Administrative Organization invited me to join, so I applied*.*

[Caregiver; family 16]

My mom invited me to come and learn together*.*

[Child; family 14]

### Theme 2: Perceptions of Learning Utility

Family members reported acquiring new skills and practices, including stress management techniques such as breathing exercises and somatic coping techniques (ie, tapping various points on the body) to help control anger and reduce stress. Caregivers also reported learning positive communication skills, including encouragement to use reason instead of emotions, speaking in a softer tone of voice, and actively listening to children’s needs. Caregivers shared that they learned about their children’s stress levels through activities such as the “stress ladder” or “thermometer,” gaining insights into children’s perspectives that adults might not have previously known. They also reported learning how to show affection and establish discipline appropriately without using violence, including techniques for hugging, praising, and setting rules in the home:

Knowing how to manage our own emotions. Knowing how to persuade children to be interested in our instructions*.*

[Caregiver; family 11]

I didn't listen to my child’s reasoning very much. After attending the training, I was able to review and listen to my child’s reasons more*.*

[Caregiver; family 6]

I learned about the activities. I'm guessing what level of expectation they [children] have, so they can use it to adjust*.*

[Caregiver; family 7]

I learn to express my stress. Then, rethink the situation or do something that helps relieve my stress*.*

[Child; family 10]

Many things, for example, calming the mind and improving mood, such as the arm raise and lower pose, which makes the mind feel better*.*

[Child; family 14]

### Theme 3: Goodness Manifesting Across Household Members

Caregivers shared insights that they became more mindful and patient and reduced scolding, yelling, and use of violence (hitting children). Instead, they started reasoning more. Children became more responsible, enacting behaviors such as helping with household chores (washing dishes, doing laundry, and ironing), studying diligently, and doing homework independently without being told. Additionally, some children showed a decrease in aggressive behavior, such as stopping or reducing bullying at school. At the family level, it was found that the atmosphere at home improved; members became closer, talked more openly, and showed more affection toward each other such as hugging or kissing cheeks. Positive changes extended to other family members even though the spouses or other parents did not attend the training. When the parent who attended the program changed their behavior, the nonattending parent was found to drink less alcohol or act less violently and be more helpful with household chores. The younger siblings who did not attend the program often followed the positive behavior of the older sibling who did participate:

My child is easier to talk to, more confident making eye contact, and more willing to share problems. Because we don't yell or scold like before, but talk things out with reason*.*

[Caregiver; family 16]

My child barely hugged me. But now, before he leaves, he comes to hug and kiss me*.*

[Caregiver; family 6]

I have tried to think that our child is just like us. So, my anger has subsided*.*

[Caregiver; family 11]

I took what I learned from the activity and told my husband. He started speaking better, so our arguments decreased*.*

[Caregiver; family 3]

I study much harder now than I used to. I've become much more responsible.... Dad drinks less alcohol, and my younger sibling helps out more*.*

[Child; family 14]

I do not tease on my friend anymore*.*

[Child; family 3]

### Theme 4: Key Facilitators of Success

Caregivers commented that the SF Program was a creative and fun activity and a family learning activity through shared experiences. Hearing problems from other families helped parents see their own strengths and weaknesses, not feel alone in solving problems, and know that they had clear and effective tools to improve the functioning of their family:

It helps parents learn the right way to teach their children, not just by forcing them, but by teaching them to learn through hands-on experience*.*

[Caregiver; family 18]

If the family is strong-minded, bad external influences cannot affect our family*.*

[Caregiver; family 11]

There was a fun game activity with my mom*.*

[Child; family 16]

### Theme 5: Recommendations for Optimization

To improve the experience of being enrolled in the SF Program and the family outcomes, caregivers stated that they would like to see activities organized regularly or continuously (eg, once a year) to prevent knowledge from fading. Participating caregivers suggested that the target group should be expanded to include more family members, such as other relatives, guardians, or siblings. Regarding the venue and time, participants shared that the activities were too short and some lacked depth. Some families suggested holding the SF Program sessions on Saturdays (as opposed to Sundays) to avoid religious obligations, and they also wanted a larger venue. They would like to see more content and training on etiquette for children, the language in the curriculum simplified, and families with specific problems selected as the target group to achieve the most noticeable changes:

I wish there were training for husbands or fathers too.... If the family training program could be done together, it would be even better*.*

[Caregiver; family 14]

I want the program to get along with the older children who have to take care of their younger siblings.... We can help each other a lot*.*

[Caregiver; family 5]

I want to do more activities with my mom*.*

[Child; family 10]

## Discussion

### Principal Findings

This study represents the evaluation of the UNODC SF Program in Thailand. Findings suggest that while the program was highly acceptable and qualitatively perceived as beneficial, the quantitative outcomes present a complex picture of family dynamics during the transition phase of behavior change. The pattern of findings observed in this study is consistent with family systems and developmental prevention theories, which emphasize the reciprocal and dynamic nature of caregiver-child interactions. Improvements in coercive parenting alongside reductions in child conduct problems suggest that even brief changes in caregiver behavior can rapidly influence child outcomes through feedback processes within the family unit. Family skills interventions that focus on caregiver stress regulation, emotional awareness, and nonviolent discipline have been shown to interrupt maladaptive interaction cycles and promote healthier relational patterns, particularly in contexts of chronic socioeconomic stress. In this way, the SF Program operates by modifying discrete behaviors and also by strengthening relational processes that support child development and family resilience within broader ecological systems [4,18,19]. Regarding parenting and family adjustment (PAFAS), the results showed a significant improvement in coercive parenting. However, a decline in positive parenting skills and relationship quality during the study period was observed. In terms of child difficulties (SDQ), parents reported significant improvements. However, social relationships showed a non–statistically significant reduction in strength. Similarly, children self-reported significant improvements in emotional and conduct behaviors. No significant changes were observed in child resilience (CYRM-R-PMK) from either the parent or child perspective.

These findings are consistent with the program’s logic model, which emphasizes stress management and nonviolent discipline. The qualitative data strongly corroborate this as caregivers reported becoming more mindful, reducing yelling, and using reasoning instead of violence in interactions with their children. Similarly, children self-reported significant improvements in emotional and conduct behaviors, suggesting that even a short 3-week intervention can interrupt the cycle of negative interaction within the family. Additionally, our study on the early implementation of the SF Program in Thailand, which assessed feedback from the trainers of SF, also illustrated this consistent outcome of improving family function [8]. Previous literature illustrates that positive parent-child relationships, especially early and middle childhood, are associated with a favorable impact on children’s behavior, avoid early-onset conduct issues, and act as a buffer against negative childhood experiences, which reduces toxic stress and improves health [20,21].

### The Divergence Between Quantitative and Qualitative Findings on Relationships

An intriguing finding in our study was the discordance between the quantitative scores for parent-child relationship and positive encouragement and the qualitative narratives. While the quantitative analysis indicated a significant increase in scores, interpreted as a decline in skills based on the instrument’s inverted scoring, the qualitative themes painted a contrasting picture. In the interviews, families reported improved atmospheres, increased affection (eg, hugging), and better communication. The discordance may be attributed to response shift bias [22]. Participants likely recalibrated their internal standards of positive parenting after the intervention, leading to stricter self-evaluation at the posttest compared to the pretest time point. This may also be viewed through the stages of competence development [23]. Participants moving from unconscious incompetence to conscious incompetence tend to judge their past and current behaviors more critically, resulting in lower postintervention scores despite actual skill acquisition [23]. The significant reduction in child conduct problems (SDQ) contrasted with the nonsignificant changes in resilience scores (CYRM-R-PMK). This suggests a distinction between acute behavioral symptoms, which respond rapidly to changed parenting strategies, and resilience, which is a complex, enduring trait requiring a longer duration or sustained intervention to manifest significant change [24]. Furthermore, qualitative insights revealed that caregivers reported uncertainty about their children’s expectations, suggesting a period of relational recalibration and heightened sensitivity rather than deterioration. These findings underscore the necessity of the mixed methods approach adopted in this study as it captured the nuanced developmental processes that quantitative tools alone might overlook [25].

Rather than reflecting program failure, the observed response shift in self-reported parenting measures may represent a meaningful indicator of learning and engagement. Response shift occurs when participants recalibrate their internal standards or conceptual understanding following exposure to an intervention, resulting in more critical self-assessment at the posttest time point despite genuine behavioral improvement [22]. In parenting interventions, increased awareness of positive parenting norms may lead caregivers to judge their behaviors more stringently over time. This process aligns with stages of competence development, in which individuals move from unconscious to conscious incompetence before achieving mastery [23]. Qualitative reports of increased patience, reflective communication, and emotional regulation suggest that caregivers were actively internalizing program content, underscoring the value of mixed methods designs for capturing developmental processes that may not be fully reflected in standardized self-report measures alone [25].

The nonsignificant resilience findings also contrast with rapid gains reported in conflict-affected settings that implemented and evaluated SF, such as Afghanistan and Iran [26,27]. This divergence likely reflects baseline severity; populations with high trauma exposure, such as those in prior SF implementations, often have lower baselines and greater room for improvement. In the nonconflict Thai cohort, where stressors were socioeconomic rather than acute, resilience building appears to be a gradual process requiring a longer intervention duration than 3 weeks to produce statistically detectable change.

### Feasibility and Cultural Adaptability

Thai communities accepted SF in the 2024 pilot, with trainers reporting that the curriculum improved family relationships and mutual understanding. The pilot’s qualitative comments revealed language obstacles. Facilitators also struggled to handle intensely tough, highly emotional familial situations that spontaneously developed during group sessions. The second phase allowed for the incorporation of brief interventions and strategies for handling difficult situations relevant to the local context, as suggested from our previous phase [8]. Thus, program materials were culturally localized before phase 2. The translated texts were heavily simplified into colloquial Thai; visual assets were replaced with culturally congruent imagery (eg, Asian faces and contextually appropriate domestic scenarios); and, most importantly, the Training for the Trainers curriculum was expanded to teach trainers how to offer targeted advice and de-escalate difficult participant interactions during live facilitation. From families’ perspective, this study confirms that the SF Program is feasible and culturally adaptable for Thailand. Qualitative feedback highlighted that the program was viewed as creative and fun, and the shared experience with other families helped reduce isolation. The motivation to join varied from community persuasion to a genuine desire to resolve family conflicts, underscoring the importance of community leaders (eg, village heads and teachers) in the recruitment process for such public health interventions. The research shows that implementing SF was feasible and had a good impact on parenting skills and the mental health of families. Because the program includes sessions for both caregivers and children independently and together, SF can enhance positive characteristics and understanding of each other and help families develop new skills in coordination [27]. Families can benefit from a wide range of positive parenting activities, such as the development of good relationships, improved communication, and stress management [28].

In Myanmar, the UNODC executed a vital implementation of the SF Program in Kachin State. This open, multisite pilot feasibility and acceptability trial enrolled 100 families using a universal approach within the camps. The program demonstrated remarkable efficacy. There was an improvement across the SDQ regarding child mental health, as well as distinct enhancements in parenting practices and parent and family adjustment skills. Crucially, the data indicated that improvements were most pronounced among caregivers and children who presented with the greatest psychological and behavioral challenges at the baseline assessment, proving the intervention’s high utility in acute humanitarian crises. The success of this trial directly advocated for the programmatic prioritization of family skills [29].

### Barriers to Resilience and Future Directions

We observed no significant changes in child resilience (CYRM-R-PMK). Resilience is a complex, multidimensional construct that likely requires a longer duration to manifest than the immediate behavior changes observed in conduct or parenting styles. To increase resilience, the intervention needs to be continuous and comprehensive [30,31]. A 3-week intervention may plant the seeds for resilience, but measurable change may require longitudinal follow-up or sustained booster sessions, as suggested by participants who requested continuous activities to prevent knowledge from fading. Additionally, a recurring theme was the need for paternal involvement. Most participating caregivers were female (59/68, 86.8%), and participants explicitly expressed a wish for husbands or fathers to attend. The predominance of female caregivers in our study reflects traditional gender roles in rural Thai society, where child-rearing is often viewed as the maternal domain [32]. However, qualitative feedback explicitly calling for paternal involvement suggests a shifting awareness within families. Future iterations of the program should specifically target fathers through gender-sensitive recruitment strategies to maximize the systemic impact on family dynamics.

Evidence from parenting intervention research indicates that father-inclusive approaches are associated with greater and more sustained improvements in child behavior and family functioning, particularly when interventions jointly address discipline practices and emotional communication [33]. Future iterations of the SF Program may benefit from gender-sensitive recruitment strategies that actively engage fathers and other caregivers, thereby enhancing the systemic and equitable impact of family skills interventions.

### Limitations

Several limitations should be noted. First, the sample size was relatively small (n=68 for quantitative assessment and n=19 for qualitative assessment) and restricted to specific provinces, limiting generalizability. Second, the absence of a control group prevents us from attributing changes solely to the SF intervention. Third, volunteer families may already possess higher motivation to improve family dynamics. The potential for response shift bias in self-reported measures suggests that future studies should include observational metrics or third-party reports to validate parenting changes. Fourth, there was a potential for recall bias in the interview session. However, we aimed to assess family behavior after the training session. Finally, the short follow-up period (12 weeks) may not capture the long-term sustainability of the skills acquired.

### Conclusions

The SF Program in Thailand demonstrates strong feasibility and promising effectiveness in reducing coercive parenting, child conduct problems, and child emotional problems. While quantitative measures of relationship quality yielded mixed results, qualitative evidence strongly supports the program’s value in fostering family bonding and emotional regulation. Scaling up this intervention with an emphasis on engaging fathers and providing long-term support could significantly benefit vulnerable families in the region.

## Abbreviations

CYRM-R-PMK: Child and Youth Resilience Measure–Revised–Person Most Knowledgeable About the Child
PAFAS: Parent and Family Adjustment Scales
SDQ: Strengths and Difficulties Questionnaire
SF: Strong Families
UNODC: United Nations Office on Drugs and Crime

## References

[1] Maitoza R (2019). Family challenges created by unemployment. J Fam Soc Work.

[2] Frounfelker RL, Miconi D, Farrar J, Brooks MA, Rousseau C, Betancourt TS (2020). Mental health of refugee children and youth: epidemiology, interventions, and future directions. Annu Rev Public Health.

[3] Jeong J, Franchett EE, Ramos de Oliveira CV, Rehmani K, Yousafzai AK (2021). Parenting interventions to promote early child development in the first three years of life: a global systematic review and meta-analysis. PLoS Med.

[4] Sanders MR, Divan G, Singhal M (2022). Scaling up parenting interventions is critical for attaining the sustainable development goals. Child Psychiatry Hum Dev.

[5] Barry MM (2001). Promoting positive mental health: theoretical frameworks for practice. Int J Ment Health Promot.

[6] Campello G, Heikkila H, Maalouf W, Israelashvili M, Romano JL (2017). The Cambridge Handbook of International Prevention Science.

[7] Haar K, El-Khani A, Molgaard V, Maalouf W, Afghanistan Field Implementation Team (2020). Strong Families: a new family skills training programme for challenged and humanitarian settings: a single-arm intervention tested in Afghanistan. BMC Public Health.

[8] Buawangpong N, El-Khani A, Aramrattana A (2025). Improving parenting skill through the Strong Families program in Thailand. Arch Public Health.

[9] O’Brien RG, Kaiser MK (1985). MANOVA method for analyzing repeated measures designs: an extensive primer. Psychol Bull.

[10] Keskin B, Aktas A, Löster T, Pavelka T (2013). The 7th International Days of Statistics and Economics Conference Proceedings.

[11] Glasgow RE, Harden SM, Gaglio B (2019). RE-AIM planning and evaluation framework: adapting to new science and practice with a 20-year review. Front Public Health.

[12] Glasgow RE, Estabrooks PA, Ory MG (2020). Characterizing evolving frameworks: issues from Esmail et al. (2020) review. Implement Sci.

[13] Sanders MR, Morawska A, Haslam DM, Filus A, Fletcher R (2014). Parenting and Family Adjustment Scales (PAFAS): validation of a brief parent-report measure for use in assessment of parenting skills and family relationships. Child Psychiatry Hum Dev.

[14] Ortuño-Sierra J, Chocarro E, Fonseca-Pedrero E, Riba SS, Muñiz J (2015). The assessment of emotional and behavioural problems: internal structure of the Strengths and Difficulties Questionnaire. Int J Clin Health Psychol.

[15] Jefferies P, McGarrigle L, Ungar M (2019). The CYRM-R: a Rasch-validated revision of the Child and Youth Resilience Measure. J Evid Based Soc Work (2019).

[16] Kang Sim DJ, Strong DR, Manzano MA, Rhee KE, Boutelle KN (2020). Evaluation of dyadic changes of parent-child weight loss patterns during a family-based behavioral treatment for obesity. Pediatr Obes.

[17] Reed M, Bedard C, Perlman CM, Browne DT, Ferro MA (2023). Family functioning and health-related quality of life in parents of children with mental illness. J Child Fam Stud.

[18] Bronfenbrenner U (1979). The Ecology of Human Development: Experiments by Nature and Design.

[19] Masten AS, Monn AR (2015). Child and family resilience: a call for integrated science, practice, and professional training. Fam Relat.

[20] Frosch CA, Schoppe-Sullivan SJ, O’Banion DD (2019). Parenting and child development: a relational health perspective. Am J Lifestyle Med.

[21] Wong PD, Wong JP, van den Heuvel M (2017). The paediatrician and middle childhood parenting. Paediatr Child Health.

[22] Howard GS (1980). Response-shift bias: a problem in evaluating interventions with pre/post self-reports. Eval Rev.

[23] Schoonenboom J, Tattersall C, Miao Y, Stefanov K, Aleksieva-Petrova A (2008). Handbook on Information Technologies for Education and Training.

[24] Ungar M, Liebenberg L (2011). Assessing resilience across cultures using mixed methods: construction of the child and youth resilience measure. J Mix Methods Res.

[25] O’Cathain A, Murphy E, Nicholl J (2007). Why, and how, mixed methods research is undertaken in health services research in England: a mixed methods study. BMC Health Serv Res.

[26] Panter-Brick C, Eggerman M, Gonzalez V, Safdar S (2009). Violence, suffering, and mental health in Afghanistan: a school-based survey. Lancet.

[27] Haar K, El-Khani A, Mostashari G, Hafezi M, Malek A, Maalouf W (2021). Impact of a brief family skills training programme (“Strong Families”) on parenting skills, child psychosocial functioning, and resilience in Iran: a multisite controlled trial. Int J Environ Res Public Health.

[28] Chen Y, Haines J, Charlton BM, VanderWeele TJ (2019). Positive parenting improves multiple aspects of health and well-being in young adulthood. Nat Hum Behav.

[29] Haar K, El-Khani A, Hawng H (2025). Implementation of a family skills programme in internally displaced people camps in Kachin State, Myanmar. Healthcare (Basel).

[30] Joyce S, Shand F, Tighe J, Laurent SJ, Bryant RA, Harvey SB (2018). Road to resilience: a systematic review and meta-analysis of resilience training programmes and interventions. BMJ Open.

[31] Unjai S, Forster EM, Mitchell AE, Creedy DK (2024). Interventions to promote resilience and passion for work in health settings: a mixed-methods systematic review. Int J Nurs Stud Adv.

[32] Liamputtong P, Yimyam S, Parisunyakul S, Baosoung C, Sansiriphun N (2002). Women as mothers: the case of Thai women in northern Thailand. Int Soc Work.

[33] Panter-Brick C, Burgess A, Eggerman M, McAllister F, Pruett K, Leckman JF (2014). Practitioner review: engaging fathers--recommendations for a game change in parenting interventions based on a systematic review of the global evidence. J Child Psychol Psychiatry.

